# Supplementary material for: Association of metformin use with risk and survival outcome of esophageal cancer in patients with diabetes: A systematic review and meta-analysis
Source: PLoS One. 2025 Jan 7;20(1):e0310687. doi: 10.1371/journal.pone.0310687 (PMC11706492; doi:10.1371/journal.pone.0310687)
Supplement: S1 Checklist — (DOCX) [file pone.0310687.s001.docx]

| **Section and Topic** | **Item #** | **Checklist item** | **Location where item is reported** |
| --- | --- | --- | --- |
| **TITLE** | | |  |
| Title | 1 | Identify the report as a systematic review. | Page 1 |
| **ABSTRACT** | | |  |
| Abstract | 2 | Provide a structured summary including, as applicable: background; objectives; data sources; study appraisal and synthesis methods; results; limitations; conclusions and implications of key findings. | Page 1 |
| **INTRODUCTION** | | |  |
| Rationale | 3 | Describe the rationale for the review in the context of existing knowledge. | Page 1-2 |
| Objectives | 4 | Provide an explicit statement of the objective(s) or question(s) the review addresses. | Page 2 |
| **METHODS** | | |  |
| Eligibility criteria | 5 | Specify the inclusion and exclusion criteria for the review and how studies were grouped for the syntheses. | Page 2-3 |
| Information sources | 6 | Specify all databases, registers, websites, organisations, reference lists and other sources searched or consulted to identify studies. Specify the date when each source was last searched or consulted. | Page 2-3 |
| Search strategy | 7 | Present the full search strategies for all databases, registers and websites, including any filters and limits used. | Page 2-3 |
| Selection process | 8 | Specify the methods used to decide whether a study met the inclusion criteria of the review, including how many reviewers screened each record and each report retrieved, whether they worked independently, and if applicable, details of automation tools used in the process. | Page 3 |
| Data collection process | 9 | Specify the methods used to collect data from reports, including how many reviewers collected data from each report, whether they worked independently, any processes for obtaining or confirming data from study investigators, and if applicable, details of automation tools used in the process. | Page 3 |
| Study risk of bias assessment | 11 | Specify the methods used to assess risk of bias in the included studies, including details of the tool(s) used, how many reviewers assessed each study and whether they worked independently, and if applicable, details of automation tools used in the process. | Page 3-4 |
| Effect measures | 12 | Specify for each outcome the effect measure(s) (e.g. risk ratio, mean difference) used in the synthesis or presentation of results. | Page 3-4 |
| Synthesis methods | 13a | Describe any methods used to synthesize results and provide a rationale for the choice(s). If meta-analysis was performed, describe the model(s), method(s) to identify the presence and extent of statistical heterogeneity, and software package(s) used. | Page 3 |
|  | 13b | Describe any sensitivity analyses conducted to assess robustness of the synthesized results. | Page 3-4 |
| Certainty assessment | 14 | Describe any methods used to assess certainty (or confidence) in the body of evidence for an outcome. | Page 4 |
| **RESULTS** | | |  |
| Study selection | 15a | Describe the results of the search and selection process, from the number of records identified in the search to the number of studies included in the review, ideally using a flow diagram. | Page 4 |
|  | 15b | Cite studies that might appear to meet the inclusion criteria, but which were excluded, and explain why they were excluded. | Page 4 |
| Study characteristics | 16 | Cite each included study and present its characteristics. | Page5- 8 |
| Risk of bias in studies | 17 | Present assessments of risk of bias for each included study. | Figure 3  Page8- 9 |
| Results of individual studies | 18 | For all outcomes, present, for each study: (a) summary statistics for each group (where appropriate) and (b) an effect estimate and its precision (e.g. confidence/credible interval), ideally using structured tables or plots. | Figure 2 Table2 Figure 4  Table3 |
| Results of syntheses | 19a | For each synthesis, briefly summarise the characteristics and risk of bias among contributing studies. | Page5-8 |
|  | 19b | Present results of all statistical syntheses conducted. If meta-analysis was done, present for each the summary estimate and its precision (e.g. confidence/credible interval) and measures of statistical heterogeneity. If comparing groups, describe the direction of the effect. | Page 9-12 Figure 2 Figure 4 |
|  | 19c | Present results of all sensitivity analyses conducted to assess the robustness of the synthesized results. | Page 9-12 |
| Certainty of evidence | 20 | Present assessments of certainty (or confidence) in the body of evidence for each outcome assessed. | Page 11 Table4 |
| **DISCUSSION** | | |  |
| Discussion | 21a | Provide a general interpretation of the results in the context of other evidence. | Page 11-12 |
|  | 21b | Discuss any limitations of the evidence included in the review. | Page 13-14 |
|  | 21c | Discuss implications of the results for practice, policy, and future research. | Page 15 |
| **OTHER INFORMATION** | | |  |
| Registration and protocol | 22 | Provide registration information for the review, including register name and registration number, or state that the review was not registered. | Page 2 |
| Support | 23 | Describe sources of financial or non-financial support for the review, and the role of the funders or sponsors in the review. | N/A |
| Competing interests | 24 | Declare any competing interests of review authors. | N/A |
| Availability of data, code and other materials | 25 | Report which of the following are publicly available and where they can be found: template data collection forms; data extracted from included studies; data used for all analyses; analytic code; any other materials used in the review. | N/A |
