# Supplementary material for: Association of metformin use with risk and survival outcome of esophageal cancer in patients with diabetes: A systematic review and meta-analysis
Source: PLoS One. 2025 Jan 7;20(1):e0310687. doi: 10.1371/journal.pone.0310687 (PMC11706492; doi:10.1371/journal.pone.0310687)
Supplement: S1 Table — (DOCX) [file pone.0310687.s002.docx]

**S2 Table. Raw data for meta-analysis.**

| Literature | Study Design | Duration of metformin use (months) | Total Sample Size | Number  of patients  (Met/ NM) | NO. of new cases of EC (Met/ NM) | Incidence rate (per 100,000 person-years) (Met/ NM) | No. of deaths among patients with EC (Met/ NM) | Mortality rate (Met/ NM) | NOS  score | Stage | Histopathology | Outcomes | Reported OR/HR  (95% CI) |
| --- | --- | --- | --- | --- | --- | --- | --- | --- | --- | --- | --- | --- | --- |
| Qiao-Li Wang, 2019, Sweden | CS | - | 4,527,633 | 5411,603/4116030 | 548/802 | 3.5 /5.3 | - | - | 9 | - | ESCC | IEC | 0.68[0.54, 0.85] |
| Chin-Hsiao Tseng, 2016, China | CS | < 21.47 | 304,229 | 16216/16216 | 16216/16216 | 25.03/50.87 | - | - | 8 | - | - | IEC | 0.56[0.33, 0.94] |
| Joseph JY Sung， 2020, China | CS | 56.4 | 289,297 | 11,365/277,932 | 9/1342 | - | - | - | 7 | - | - | IEC | 0.27[0.12, 0.59] |
| Konstantinos K. Tsilidis, 2014, UK | CS | 12 | 95,820 | 51484/18264 | - | - | - | - | 7 | - | - | IEC | 1.05[0.71, 1.56] |
| Meei-Shyuan Lee， 2011, China | CS | 24 | 480,984 | 11212/4193 | 21/6 | 47.8/40.3 | - | - | 7 | - | - | IEC | 0.44[0.07, 2.61] |
| Harvey J. Murff, 2018, USA | CS | 24 | 84,434 | 42217/42217 | 42/35 | 50/50 | - | - | 8 | - | - | IEC | 0.99[0.63, 1.55] |
| Roy G. de Jong, 2017, Netherlands | CS | - | 57,114 | 37215/19899 | - | 376/457 | - | - | 8 | - | - | IEC | 0.90[0.48, 1.67] |
| Rikje Ruiter, 2012, Netherlands | CS | 12 | 85,289 | 52,698/32,591 | 45/46 | 30/30 | - | - | 9 | - | - | IEC | 0.90 [0.82, 0.97] |
| Tak Kyu Oh, 2019, South Korea | CS | 9.6 | 66,627 | 29974/36653 | - | - | - | - | 8 | - | - | IEC | 0.38 [0.13, 1.13] |
| Claudia Becker, 2013, UK | CCS | ＞60 | 4,070 | 2040/2030 | 197/173 | - | - | - | 7 | - | - | IEC | 1.11[0.79, 1.54] |
| Francesca Valent, 2015, Italian | CS | - | 109255 | 63119/75402 | - | - | - | - | 7 | - | - | IEC | 0.98[0.98,1.00] |
| Kao-Chi Cheng， 2012, China | CCS | - | 279 | 231/48 | 52/5 | - | - | - | 7 | - | - | IEC | 2.84[0.99,8.18] |
| L. Van De Voorde, 2015，Netherlands | CS | - | 196 | 19/177 | - | - | 4/89 | 21%/50.3% | 6 | T3 (68%) N0-N1 (80%) | Adenocarcinoma (78.1%) | OS | 0.35 [0.13, 0.97] |
| Huang-He He, 2020， China | CS | - | 619 | 485/134 | - | - | 335/104 | 69.07%/77.61% | 7 | II-III (77.7%) | ESCC (100%) | OS | 0.89 [0.80, 0.99] |
| L. E. A. M. M. Spierings, 2015，Netherlands | CS | - | 43 | 32/11 |  | - | - |  | 7 | T3(77.7%) N1-N2(71.1%) | Adenocarcinoma (68.3%) | OS | 0.49 [0.08, 2.92] |
| Qiaoli Wang, 2023，Sweden | CS | 12 | 852 | 473/379 |  | - | 378/344 | 79.9%/90.7% | 8 | III-IV (59.3%) | Adenocarcinoma (57.6%) ESCC (37.0%) | OS | 0.86[0.75, 1.00]  Adenocarcinoma 0.88（0.74-1.05）  ESCC 0.85 (0.64 to 1.14) |

ESCC, Esophageal squamous cell carcinoma; Met, Metformin; NM, Non-metformin; CS, Cohort study; CCS, Case control study; IEC, Incidence of esophageal cancer; OS, Overall survival; NA, Not available; EC, Esophageal cancer

| Literature | Comparators | Age (Mean years) | BMI (Mean) | Time-related biases | Adjusted by BMI | Adjusted by smoking | Treatment | Name of data extractors | Date of data extraction | Whether meet inclusion conditions |
| --- | --- | --- | --- | --- | --- | --- | --- | --- | --- | --- |
| Qiao-Li Wang, 2019, Sweden | NM | 59 |  | No | No | Yes | Statins/ aspirin | Hui Xie | August 15, 2023 | Yes |
| Chin-Hsiao Tseng, 2016, China | NM | 59 |  | Yes | No | No |  | Muhan Li | September 6, 2023 | Yes |
| Joseph JY Sung， 2020, China | NM | ＞60 |  | No | No | No | Aspirin | Muhan Li | September 6, 2023 | Yes |
| Konstantinos K. Tsilidis, 2014, UK | Sulfonylurea | ＞60 | ＞30 | Yes | Yes | Yes | Statins/ aspirin | Hui Xie | August 15, 2023 | Yes |
| Meei-Shyuan Lee， 2011, China | NM | ＜60 |  | No | No | No |  | Muhan Li | September 6, 2023 | Yes |
| Harvey J. Murff, 2018, USA | Sulfonylurea | 62 | 30.7 | No | Yes | Yes |  | Muhan Li | September 6, 2023 | Yes |
| Roy G. de Jong, 2017, Netherlands | NM | 63.5 |  | No | No | No | Statins/ aspirin/ insulin | Muhan Li | September 6, 2023 | Yes |
| Rikje Ruiter, 2012, Netherlands | Sulfonylurea | 61.8 |  | Yes | No | No |  | Muhan Li | September 9, 2023 | Yes |
| Tak Kyu Oh, 2019, South Korea | NM | 60.5 |  | No | No | No | Insulin | Muhan Li | September 10, 2023 | Yes |
| Claudia Becker, 2013, UK | NM | 69.1 |  | No | Yes | Yes |  | Muhan Li | September 10, 2023 | Yes |
| Francesca Valent, 2015, Italian | NM | - |  | Yes | No | No |  | Hui Xie | August 25, 2023 | Yes |
| Kao-Chi Cheng， 2012, China | NM | 60.9 |  | Yes | No | No |  | Hui Xie | August 27, 2023 | Yes |
| L. Van De Voorde, 2015，Netherlands | NM | 64 | ＜30 |  | No | No | Surgery/ Surgery + NC | Hui Xie | August 27, 2023 | Yes |
| Huang-He He, 2020， China | NM | 59.94 |  |  | Yes | Yes | Surgery | Hui Xie | August 27, 2023 | Yes |
| L. E. A. M. M. Spierings, 2015，Netherlands | NM | 66 | ＜30 |  | No | No | Surgery/ Surgery + NC/ statins | Hui Xie | August 27, 2023 | Yes |
| Qiaoli Wang, 2023，Sweden | NM | 70 |  |  | No | No | Without Surgery/ Surgery/ statins | Muhan Li | September 10, 2023 | Yes |

NM, Non-metformin; BMI, body mass index; NC, neoadjuvant chemoradiotherapy
