# Supplementary material for: Association of metformin use with risk and survival outcome of esophageal cancer in patients with diabetes: A systematic review and meta-analysis
Source: PLoS One. 2025 Jan 7;20(1):e0310687. doi: 10.1371/journal.pone.0310687 (PMC11706492; doi:10.1371/journal.pone.0310687)
Supplement: S2 Table — (DOC) [file pone.0310687.s003.doc]

S4 Table. Quality assessment of retrospective studies using the Newcastle-Ottawa scale

| **First author, year** | **Selection** | | | |  | **Comparability** | |  | **Outcome** | | | **Score** |
| --- | --- | --- | --- | --- | --- | --- | --- | --- | --- | --- | --- | --- |
|  | Item 1 | Item 2 | Item 3 | Item 4 |  | Item 5 | Item 6 |  | Item 7 | Item 8 | Item 9 |  |
| Qiao-Li Wang, 2019 | * | * | * | * |  | * | * |  | * | - | * | 8 |
| Chin-Hsiao Tseng, 2016 | * | * | * | * |  | * | * |  | * | - | * | 8 |
| Joseph JY Sung， 2020 | * | * | * | * |  | * | * |  | * | - | - | 7 |
| Konstantinos K. Tsilidis, 2014 | * | * | * | * |  | * | * |  | * | - | * | 8 |
| Meei-Shyuan Lee， 2011 | * | * | * | * |  | * | * |  | * | - | - | 7 |
| Harvey J. Murff, 2018 | - | - | * | * |  | * | * |  | * | - | * | 6 |
| Roy G. de Jong, 2017 | * | * | * | * |  | * | * |  | * | - | * | 8 |
| Rikje Ruiter, 2012 | * | * | * | * |  | * | * |  | * | - | - | 7 |
| Tak Kyu Oh, 2019 | * | * | * | * |  | * | * |  | * | - | - | 7 |
| Claudia Becker, 2013 | * | * | * | - |  | * | * |  | * | - | - | 6 |
| Francesca Valent, 2015 | * | * | * | * |  | * | * |  | * | - | - | 7 |
| Kao-Chi Cheng， 2012 | * | * | * | - |  | * | * |  | * | - | - | 6 |
| L. Van De Voorde, 2015 | * | * | * | * |  | - | - |  | * | - | * | 6 |
| Huang-He He, 2020 | * | * | * | * |  | * | * |  | * | - | * | 8 |
| L. E. A. M. M. Spierings, 2015 | * | * | * | * |  | - | - |  | * | - | * | 6 |
| Qiaoli Wang, 2023 | * | * | * | * |  | * | * |  | * | - | * | 8 |

–, zero point; *, one point. Item 1, representativeness of the exposed cohort; item 2, selection of the non-exposed cohort; item 3, ascertainment of exposure; item 4, demonstration that outcome of interest was not present at start of study; item 5, comparability of cohorts on the basis of the design (study controls for the most important factor); item 6, comparability of cohorts on the basis of the design (study controls for other additional factor); item 7, assessment of outcome; item 8, follow-up long enough for outcomes to occur; item 9, adequacy of follow-up of cohorts.
