# Supplementary material for: Association of metformin use with risk and survival outcome of esophageal cancer in patients with diabetes: A systematic review and meta-analysis
Source: PLoS One. 2025 Jan 7;20(1):e0310687. doi: 10.1371/journal.pone.0310687 (PMC11706492; doi:10.1371/journal.pone.0310687)
Supplement: S3 Table — (DOCX) [file pone.0310687.s004.docx]

**S3 Table. Articles with reasons for exclusion.**

| No. | Reference | Reason for exclusion |
| --- | --- | --- |
| 1 | Sekino N, Kano M, Matsumoto Y, Sakata H, Akutsu Y, Hanari N, et al. Antitumor effects of metformin are a result of inhibiting nuclear factor kappa B nuclear translocation in esophageal squamous cell carcinoma. Cancer Sci. 2018;109(4):1066-74. http://doi.org/10.1111/cas.13523 | Cell experiment |
| 2 | Manno V, Lentini N, Chirico A, Perticone M, Anastasio L. Acute esophageal necrosis (black esophagus): a case report and literature review. Acta Diabetol. 2017;54(11):1061-3. http://doi.org/10.1007/s00592-017-1028-4 | Review |
| 3 | Tunio MA, AlAsiri M, Riaz K. Adenocarcinoma of oesophagus metastasising to the subcutaneous soft tissue. Arab J Gastroenterol. 2013;14(3):133-4. http://doi.org/10.1016/j.ajg.2013.05.005 | Case Report |
| 4 | Hefetz-Sela S, Scherer PE. Adipocytes: Impact on tumor growth and potential sites for therapeutic intervention. Pharmacology and Therapeutics. 2013;138(2):197-210. http://doi.org/10.1016/j.pharmthera.2013.01.008 | Review |
| 5 | Sekino N, Kano M, Matsumoto Y, Sakata H, Akutsu Y, Hanari N, et al. Antitumor effects of metformin are a result of inhibiting nuclear factor kappa B nuclear translocation in esophageal squamous cell carcinoma. Cancer Sci. 2018;109(4):1066-74. http://doi.org/10.1111/cas.13523 | Cell experiment |
| 6 | Joo MK, Park J, Chun HJ. Additional Benefits of Routine Drugs on Gastrointestinal Cancer: Statins, Metformin, and Proton Pump Inhibitors. Digest Dis. 2017;36(1):1-14. http://doi.org/10.1159/000480149 | Review |
| 7 | Chen CJ, Chiu WC, Tseng YH, Lin CM, Yang HY, Yang YH, et al. Aristolochic acid and the risk of cancers in patients with type 2 diabetes: Nationwide population-based cohort study. Phytomedicine. 2022;99. http://doi.org/10.1016/j.phymed.2022.154023 | Population not relevant |
| 8 | Chaudhary SK, Sandasi M, Makolo F, van Heerden FR, Viljoen AM. Aspalathin: a rare dietary dihydrochalcone from Aspalathus linearis (rooibos tea). Phytochem Rev. 2021;20(6):1161-92. http://doi.org/10.1007/s11101-021-09741-9 | Unrelated drug treatment |
| 9 | Seo SI, Park CH, Kim TJ, Bang CS, Kim JY, Lee KJ, et al. Aspirin, metformin, and statin use on the risk of gastric cancer: A nationwide population-based cohort study in Korea with systematic review and meta-analysis. Cancer Med-Us. 2022;11(4):1217-31. http://doi.org/10.1002/cam4.4514 | Meta-analysis |
| 10 | Kasiri K, Sherwin CMT, Rostamian S, Heidari-Soureshjani S. Assessment of the Relationship Between Gastric-Acid Suppressants and the Risk of Esophageal Adenocarcinoma: A Systematic Review and Meta-Analysis. Current Therapeutic Research - Clinical and Experimental. 2023;98. http://doi.org/10.1016/j.curtheres.2023.100692 | Meta-analysis |
| 11 | Fattouh M, Chang GY, Ow TJ, Shifteh K, Rosenblatt G, Patel VM, et al. Association between pretreatment obesity, sarcopenia, and survival in patients with head and neck cancer. Head Neck-J Sci Spec. 2019;41(3):707-14. http://doi.org/10.1002/hed.25420 | Population not relevant |
| 12 | Gupta SC, Sung B, Prasad S, Webb LJ, Aggarwal BB. Cancer drug discovery by repurposing: Teaching new tricks to old dogs. Trends Pharmacol Sci. 2013;34(9):508-17. http://doi.org/10.1016/j.tips.2013.06.005 | Review |
| 13 | Albini A, Tosetti F, Li VW, Noonan DM, Li WW. Cancer prevention by targeting angiogenesis. Nat Rev Clin Oncol. 2012;9(9):498-509. http://doi.org/10.1038/nrclinonc.2012.120 | Review |
| 14 | Seebauer W. Cancer, diabetes and nutrition: Results of the European Prospective Investigation into Cancer and Nutrition study. Kim - Komplementare Und Integrative Medizin, Artztezeitschrift Fur Naturheilverfahren. 2009;50(4):19-26. http://doi.org/10.1016/j.kim.2009.02.014 | Population not relevant |
| 15 | Badila E, Japie C, Vrabie AM, Badila A, Georgescu A. Cardiovascular Disease as a Consequence or a Cause of Cancer: Potential Role of Extracellular Vesicles. Biomolecules. 2023;13(2). http://doi.org/10.3390/biom13020321 | Review |
| 16 | Gokani S, Bhatt LK. Caveolin-1: A Promising Therapeutic Target for Diverse Diseases. Curr Mol Pharmacol. 2022;15(5):701-15. http://doi.org/10.2174/1874467214666211130155902 | Review |
| 17 | Zanders MMJ, Haak HR, van Herk-Sukel MPP, Herings RMC, van de Poll-Franse LV, Johnson JA. Changes in glucose-lowering drug use before and after cancer diagnosis in patients with diabetes. Diabetes and Metabolism. 2018;44(1):22-9. http://doi.org/10.1016/j.diabet.2017.08.004 | Unrelated drug treatment |
| 18 | Rohm M, Herzig S, Schafmeier T. Chubby and healthy? - The contribution of overweight to carcinogenesis and implications for the treatment. Deutsche Zeitschrift Fur Onkologie. 2014;46(4):167-74. http://doi.org/10.1055/s-0033-1357707 | Review |
| 19 | Yazlcl D, Yaplcl Eser H, Klylcl S, Sancak S, Sezer H, Uygur M, et al. Clinical Impact of Glucagon-Like Peptide-1 Receptor Analogs on the Complications of Obesity. Obesity Facts. 2023;16(2):149-63. http://doi.org/10.1159/000526808 | Review |
| 20 | Chen S, Zhu X, Lai X, Xiao T, Wen A, Zhang J. Combined cancer therapy with non-conventional drugs: All roads lead to AMPK. Mini-Rev Med Chem. 2014;14(8):642-54. | Review |
| 21 | Moayyedi P, El-Serag HB. Current Status of Chemoprevention in Barrett's Esophagus. Gastrointestinal Endoscopy Clinics of North America. 2021;31(1):117-30. http://doi.org/10.1016/j.giec.2020.08.008 | Review |
| 22 | Bolinder J, Ljunggren Ö, Johansson L, Wilding J, Langkilde AM, Sjöström CD, et al. Dapagliflozin maintains glycaemic control while reducing weight and body fat mass over 2 years in patients with type 2 diabetes mellitus inadequately controlled on metformin. 2014;16(2):159-69. http://doi.org/10.1111/dom.12189 | Unrelated drug treatment |
| 23 | Wang T, Ning G, Bloomgarden Z. Diabetes and cancer relationships. J Diabetes. 2013;5(4):378-90. http://doi.org/10.1111/1753-0407.12057 | Review |
| 24 | Giovannucci E, Harlan DM, Archer MC, Bergenstal RM, Gapstur SM, Habel LA, et al. Diabetes and cancer: A consensus report. Diabetes Care. 2010;33(7):1674-85. http://doi.org/10.2337/dc10-0666 | Review |
| 25 | Habib SL, Rojna M. Diabetes and risk of cancer. Isrn Oncology. 2013;1(1). http://doi.org/10.1155/2013/583786 | Review |
| 26 | Escaja CR, Navascues CA, Gonzalez-Dieguez L, Cadahia V, Varela M, De Jorge MA, et al. DIABETES IS NOT ASSOCIATED WITH AN INCREASED RISK OF HEPATOCELLULAR CARCINOMA IN PATIENTS WITH ALCOHOLIC, HBV OR HCV LIVER CIRRHOSIS. J Hepatol. 2016;642:S322. http://doi.org/10.1016/S0168-8278(16)00440-2 | Population not relevant |
| 27 | Zheng X, Ma X, Deng H, Zha P, Zhou J, Wang R, et al. Diabetes mellitus and survival of esophageal cancer patients after esophagectomy: a systematic review and meta-analysis. Dis Esophagus. 2020;33(DOZ0982). http://doi.org/10.1093/dote/doz098 | Meta-analysis |
| 28 | Gallagher EJ, Leroith D. Diabetes, antihyperglycemic medications and cancer risk: Smoke or fire? Current Opinion in Endocrinology, Diabetes and Obesity. 2013;20(5):485-94. http://doi.org/10.1097/01.med.0000433065.16918.83 | Review |
| 29 | Murthy N, Mukherjee S, Ray G, Ray A. Dietary factors and cancer chemoprevention: An overview of obesity-related malignancies. J Postgrad Med. 2009;55(1):45-54. http://doi.org/10.4103/0022-3859.43549 | Review |
| 30 | Gulfidan G, Beklen H, Sinha I, Kucukalp F, Caloglu B, Esen I, et al. Differential Protein Interactome in Esophageal Squamous Cell Carcinoma Offers Novel Systems Biomarker Candidates with High Diagnostic and Prognostic Performance. Omics a Journal of Integrative Biology. 2021;25(8):495-512. http://doi.org/10.1089/omi.2021.0085 | Cell experiment |
| 31 | Golozar A, Liu S, Lin JA, Peairs K, Yeh HC. Does Metformin Reduce Cancer Risks? Methodologic Considerations. Curr Diabetes Rep. 2016;16(1):1-11. http://doi.org/10.1007/s11892-015-0697-z | Review |
| 32 | Dawson JC, Warchal SJ, Carragher NO. Drug Screening Platforms and RPPA.; 2019. p. 203-26. | Review |
| 33 | Conroy RT, Siddiqi B. Dyspepsia. Primary Care - Clinics in Office Practice. 2007;34(1):99-108. http://doi.org/10.1016/j.pop.2007.02.001 | Review |
| 34 | López-Urrutia E, Padilla-Benavides T, Pérez-Plasencia C, Campos-Parra AD. Editorial: Repurposed Drugs Targeting Cancer Signaling Pathways: Dissecting New Mechanism of Action Through In Vitro and In Vivo Analyses. Front Oncol. 2021;11. http://doi.org/10.3389/fonc.2021.773429 | Review |
| 35 | Kim YI, Kim YA, Kim HJ, Kim SH, Hwangbo Y, Kim JG, et al. Effect of helicobacter pylori treatment on the long-term mortality in patients with type 2 diabetes. Korean J Intern Med. 2021;36(3):584-95. http://doi.org/10.3904/kjim.2019.428 | Unrelated drug treatment |
| 36 | Rao M, Gao C, Guo M, Law BYK, Xu Y. Effects of metformin treatment on radiotherapy efficacy in patients with cancer and diabetes: A systematic review and meta-analysis. Cancer Manag Res. 2018;10:4881-90. http://doi.org/10.2147/CMAR.S174535 | Meta-analysis |
| 37 | Liu B, Liu D, Zhao L, Kong L, Ahmed MR, Li D, et al. Endoscopic gastric mucosal ablation (EGMA) for weight loss: The first human case report. United Eur Gastroent. 2018;6(8):A538-9. http://doi.org/10.1177/2050640618792819 | Case report |
| 38 | Fay JR, Steele V, Crowell JA. Energy homeostasis and cancer prevention: The AMP-activated protein kinase. Cancer Prev Res. 2009;2(4):301-9. http://doi.org/10.1158/1940-6207.CAPR-08-0166 | Review |
| 39 | Thrift AP. Esophageal Adenocarcinoma: The Influence of Medications Used to Treat Comorbidities on Cancer Prognosis. Clin Gastroenterol H. 2015;13(13):2225-32. http://doi.org/10.1016/j.cgh.2015.03.028 | Review |
| 40 | Home PD, Kahn SE, Jones NP, Noronha D, Beck-Nielsen H, Viberti G. Experience of malignancies with oral glucose-lowering drugs in the randomised controlled ADOPT (A Diabetes Outcome Progression Trial) and RECORD (Rosiglitazone Evaluated for Cardiovascular Outcomes and Regulation of Glycaemia in Diabetes) clinical trials. Diabetologia. 2010;53(9):1838-45. http://doi.org/10.1007/s00125-010-1804-y | Unrelated drug treatment |
| 41 | de Jong RGPJ, Peeters PJHL, Burden AM, de Bruin ML, Haak HR, Masclee AAM, et al. Gastrointestinal cancer incidence in type 2 diabetes mellitus; results from a large population-based cohort study in the UK. Cancer Epidemiol. 2018;54:104-11. http://doi.org/10.1016/j.canep.2018.04.008 | Unrelated drug treatment |
| 42 | Krstic MN, Mijac DD, Popovic DD, Markovic AP, Milosavljević T. General Aspects of Primary Cancer Prevention. Digest Dis. 2019;37(5):406-15. http://doi.org/10.1159/000497191 | Review |
| 43 | Krishnan A, Hadi Y, Hutson WR, Thakkar S, Singh S. Glucagon-Like Peptide 1-Based Therapies and Risk of Pancreatic Cancer in Patients with Diabetes and Obesity. Pancreas. 2022;51(10):1398-403. http://doi.org/10.1097/MPA.0000000000002197 | Population not relevant |
| 44 | Piatkiewicz P, Czech A. Glucose Metabolism Disorders and the Risk of Cancer. Arch Immunol Ther Ex. 2011;59(3):215-30. http://doi.org/10.1007/s00005-011-0119-0 | Review |
| 45 | Hochwald JS, Zhang J. Glucose oncometabolism of esophageal cancer. Anti-Cancer Agent Me. 2017;17(3):385-94. http://doi.org/10.2174/1871520616666160627092716 | Review |
| 46 | Lopes S, Figueiredo P, Amaro P, Freire P, Alves S, Cipriano MA, et al. Glycogenic acanthosis of the esophagus: An unusually endoscopic appearance. Rev Esp Enferm Dig. 2010;102(5):341-2. http://doi.org/10.4321/S1130-01082010000500013 | Case Report |
| 47 | Shanazarov NA, Seidalin NK, Khalirakhmanov AF, Sharafeev AZ, Gatuyatullina GD, Zinchenko SV. Heart failure in oncological patients. International Journal of Pharmaceutical Research. 2020;12(3):2968-73. http://doi.org/10.31838/ijpr/2020.12.03.419 | Review |
| 48 | Han S, Yadlapati R, Simon VC, Ezekwe E, Early DS, Kushnir V, et al. HIGHER DEGREES OF DYSPLASIA ARE ASSOCIATED WITH POOR QUALITY OF LIFE IN BARRETT'S ESOPHAGUS PATIENTS REFERRED FOR ENDOSCOPIC ERADICATION THERAPY: RESULTS FROM A MULTICENTER COHORT STUDY. Gastroenterology. 2018;1541(6):S335-6. | Population not relevant |
| 49 | Kode S, Amro A, James D, Vacheron A, Solomon S. How does metformin produce its anti-cancer effects? J Invest Med. 2020;68(2):485. http://doi.org/10.1136/jim-2020-SRM.125 | Review |
| 50 | He LR, Qiao W, Liao ZX, Komaki R, Ho L, Hofstetter WL, et al. Impact of comorbidities and use of common medications on cancer and non-cancer specific survival in esophageal carcinoma. Bmc Cancer. 2015;15:1095. http://doi.org/10.1186/s12885-015-1095-2 | Unrelated treatment |
| 51 | de Jong RGPJ, Burden AM, de Kort S, van Herk-Sukel MPP, Vissers PAJ, Janssen PKC, et al. Impact of detection bias on the risk of gastrointestinal cancer and its subsites in type 2 diabetes mellitus. Eur J Cancer. 2017;79:61-71. http://doi.org/10.1016/j.ejca.2017.03.039 | Unrelated treatment |
| 52 | Lacroix O, Couttenier A, Vaes E, Cardwell CR, De Schutter H, Robert A. Impact of metformin on gastric adenocarcinoma survival: A Belgian population based study. Cancer Epidemiol. 2018;53:149-55. http://doi.org/10.1016/j.canep.2018.02.001 | Population not relevant |
| 53 | Wilder E, Poles J, Yang B, Popov V. Impact of Metformin Use on Esophageal Cancer Recurrence and Survival: A Systematic Review and Meta-Analysis. Am J Gastroenterol. 2018;113S:S211-2. | Meta-Analysis |
| 54 | Honjo S, Kawasaki Y, Hamamoto Y, Mori K, Ikeda H, Wada Y, et al. Incidence and Type of Cancer in Japanese Subjects with Type 2 Diabetes. Diabetes. 2011;601:A651. | Unrelated treatment |
| 55 | Oh S, Lee J, Hong YS, Kim K. Increased risk of cardiovascular disease associated with diabetes among adult cancer survivors: a population-based matched cohort study. Eur J Prev Cardiol. 2023;30(8):670-9. http://doi.org/10.1093/eurjpc/zwad046 | Population not relevant |
| 56 | Cho O, Oh YT, Chun M, Noh OK, Heo J. Influence of insulin therapy for type 2 diabetes mellitus on cancer incidence. Add to Collection. J Clin Oncol. 2017;35(15). | Unrelated drug treatment |
| 57 | Shafaee A, Dastyar DZ, Islamian JP, Hatamian M. Inhibition of tumor energy pathways for targeted esophagus cancer therapy. Metabolism. 2015;64(10):1193-8. http://doi.org/10.1016/j.metabol.2015.07.005 | Review |
| 58 | Arcidiacono B, Iiritano S, Nocera A, Possidente K, Nevolo MT, Ventura V, et al. Insulin resistance and cancer risk: An overview of the pathogenetic mechanisms. Experimental Diabetes Research. 2012;2012. http://doi.org/10.1155/2012/789174 | Review |
| 59 | Gallagher EJ, LeRoith D. Insulin, insulin resistance, obesity, and cancer. Curr Diabetes Rep. 2010;10(2):93-100. http://doi.org/10.1007/s11892-010-0101-y | Review |
| 60 | Fan G, Hu D, Zhang X, Peng F, Lin X, Chen G, et al. Interaction Between Prediabetes and the ABO Blood Types in Predicting Postsurgical Esophageal Squamous Cell Carcinoma-Specific Mortality: The FIESTA Study. Front Oncol. 2018;8(461). http://doi.org/10.3389/fonc.2018.00461 | Unrelated drug treatment |
| 61 | Wright CM, Shastri AA, Bongiorno E, Palagani A, Rodeck U, Simone NL. Is host metabolism the missing link to improving cancer outcomes? Cancers. 2020;12(9):1-20. http://doi.org/10.3390/cancers12092338 | Review |
| 62 | Jindal T, Sarwal A, Pawar P, Dhanalakshmi M, Subedi N. Laparoscopic management of isolated metachronous adrenal metastases in a patient with esophageal cancer: a case report. Journal of Medical Case Reports. 2021;15(1). http://doi.org/10.1186/s13256-021-02849-8 | Case report |
| 63 | Shuvalov O, Daks A, Fedorova O, Petukhov A, Barlev N. Linking metabolic reprogramming, plasticity and tumor progression. Cancers. 2021;13(4):1-25. http://doi.org/10.3390/cancers13040762 | Review |
| 64 | Labochka D, Moszczuk B, Kukwa W, Szczylik C, Czarnecka AM. Mechanisms through which diabetes mellitus influences renal cell carcinoma development and treatment: A review of the literature. Int J Mol Med. 2016;38(6):1887-94. http://doi.org/10.3892/ijmm.2016.2776 | Review |
| 65 | Mayer D, Chantelau E. Meeting Report: 3rd International Workshop on Insulin & Cancer Heidelberg, Germany, October 30-31, 2010. Pediatric Endocrinology Reviews. 2011;8(3):226-7. | Conference |
| 66 | Extermann M. Metabolic Syndrome, Aging, and Cancer. Critical Reviews in Oncogenesis. 2013;18(6, Sp. Iss. SI):515-29. | Review |
| 67 | Rizos CV, Elisaf MS. Metformin and cancer. Eur J Pharmacol. 2013;705(1-3):96-108. http://doi.org/10.1016/j.ejphar.2013.02.038 | Review |
| 68 | Skuli SJ, Alomari S, Gaitsch H, Bakayoko A, Skuli N, Tyler BM. Metformin and Cancer, an Ambiguanidous Relationship. Pharmaceuticals-Base. 2022;15(5). http://doi.org/10.3390/ph15050626 | Review |
| 69 | Samsuri NAB, Leech M, Marignol L. Metformin and improved treatment outcomes in radiation therapy - A review. Cancer Treat Rev. 2017;55:150-62. http://doi.org/10.1016/j.ctrv.2017.03.005 | Review |
| 70 | Zhang K, Bai P, Dai H, Deng Z. Metformin and risk of cancer among patients with type 2 diabetes mellitus: A systematic review and meta-analysis. Prim Care Diabetes. 2021;15(1):52-8. http://doi.org/10.1016/j.pcd.2020.06.001 | Meta-analysis |
| 71 | Tseng C. Metformin and risk of developing nasopharyngeal cancer in patients with type 2 diabetes mellitus. Metabolism. 2018;85:223-6. http://doi.org/10.1016/j.metabol.2018.04.009 | Population not relevant |
| 72 | Wynn A, Vacheron A, Zuber J, Solomon SS. Metformin Associated With Increased Survival in Type 2 Diabetes Patients With Pancreatic Cancer and Lymphoma. Am J Med Sci. 2019;358(3):200-3. http://doi.org/10.1016/j.amjms.2019.06.002 | Population not relevant |
| 73 | Lee SH, Jin S, Lee HS, Ryu JS, Lee JJ. Metformin discontinuation less than 72 h is suboptimal for F-18 FDG PET/CT interpretation of the bowel. Ann Nucl Med. 2016;30(9):629-36. http://doi.org/10.1007/s12149-016-1106-7 | Population not relevant |
| 74 | Chen S, Gan D, Lin S, Zhong Y, Chen M, Zou X, et al. Metformin in aging and aging-related diseases: clinical applications and relevant mechanisms. Theranostics. 2022;12(6):2722-40. http://doi.org/10.7150/thno.71360 | Review |
| 75 | Morales DR, Morris AD. Metformin in cancer treatment and prevention.; 2015. p. 17-29. | Review |
| 76 | Vacheron A, Wynn A, Zuber J, Solomon S. Metformin increases survival in T2DM patients with pancreatic cancer and lymphoma. J Invest Med. 2019;67(2):624-5. http://doi.org/10.1136/jim-2018-000974.690 | Population not relevant |
| 77 | Fan H, Zou Z, Yu X, Guo L, Jiang W, Lu SH. Metformin represses esophageal carcinogenesis in NMBzA-treated rat model through inhibiting AMPK/mTOR and Stat3 signaling pathways. Cancer Res. 2017;77(13). http://doi.org/10.1158/1538-7445.AM2017-1266 | Animal experiment |
| 78 | Franciosi M, Lucisano G, Lapice E, Strippoli GF, Pellegrini F, Nicolucci A. Metformin therapy and risk of cancer in patients with type 2 diabetes: systematic review. Plos One. 2013;8(8):e71583. http://doi.org/10.1371/journal.pone.0071583 | Review |
| 79 | Skinner HD, McCurdy MR, Echeverria AE, Lin SH, Welsh JW, O'Reilly MS, et al. Metformin use and improved response to therapy in esophageal adenocarcinoma. Acta Oncol. 2013;52(5):1002-9. http://doi.org/10.3109/0284186X.2012.718096 | Unable to get the outcome indicate |
| 80 | Zheng J, Xie S, Santoni G, Lagergren J. Metformin use and risk of gastric adenocarcinoma in a Swedish population-based cohort study. Brit J Cancer. 2019;121(10):877-82. http://doi.org/10.1038/s41416-019-0598-z | Population not relevant |
| 81 | Agrawal S, Agrawal A, Makhijani N, Patel P, Markert R, Diedrich W. Metformin use and the risk of esophageal adenocarcinoma in patients with barrett's esophagus. Am J Gastroenterol. 2013;108:S14. http://doi.org/10.1038/ajg.2013.261 | Population not relevant |
| 82 | Agrawal S, Patel P, Agrawal A, Makhijani N, Markert R, Deidrich W. Metformin use and the risk of esophageal cancer in Barrett esophagus. South Med J. 2014;107(12):774-9. http://doi.org/10.14423/SMJ.0000000000000212 | Population not relevant |
| 83 | McAvoy SA, Soh HE, Komaki R, Cox JD, Welsh JW, Guerrero TM, et al. Metformin Use Does Not Affect Chemoradiotherapy-Associated Toxicity. Oncology-Basel. 2014;28(1S). | Purpose of the study not relevant |
| 84 | Wu H, Huang D, Zhou H, Sima X, Wu Z, Sun Y, et al. Metformin: A promising drug for human cancers. Oncol Lett. 2022;24(2041). http://doi.org/10.3892/ol.2022.13325 | Review |
| 85 | Rattan R, Ali Fehmi R, Munkarah A. Metformin: An emerging new therapeutic option for targeting cancer stem cells and metastasis. J Oncol. 2012. http://doi.org/10.1155/2012/928127 | Review |
| 86 | Nar A. Metformin: Hype or Hope for Cancer. Uhod-Uluslar Hematol. 2017;27(4):258-66. http://doi.org/10.4999/uhod.171870 | Review |
| 87 | Ma J, Wang J, Zhao Q, Yu X, Li H, Wang F, et al. Metformin: Moving the cheese for tumor? Int J Clin Exp Med. 2017;10(11):15064-70. | Review |
| 88 | Markowicz-Piasecka M, Huttunen KM, Mikiciuk-Olasik E, Mateusiak A, Sikora J. Metformin-from anti-diabetic drug to anti-cancer drug. Acta Poloniae Pharmaceutica - Drug Research. 2018;75(1):3-18. | Review |
| 89 | Hursting SD. Minireview: The Year in Obesity and Cancer. Molecular Endocrinology. 2012;26(12):1961-6. http://doi.org/10.1210/me.2012-1283 | Review |
| 90 | Hussain I, Jin RR, Baum HBA, Greenfield JR, Devery S, Xing C, et al. Multisystem progeroid syndrome with lipodystrophy, cardiomyopathy, and nephropathy due to an LMNA p.R349W variant. J Endocr Soc. 2020;4(10). http://doi.org/10.1210/jendso/bvaa104 | Population not relevant |
| 91 | Tichy EM, Hoffman JM, Suda KJ, Rim MH, Tadrous M, Cuellar S, et al. National trends in prescription drug expenditures and projections for 2022. Am J Health-Syst Ph. 2022;79(14):1158-72. http://doi.org/10.1093/ajhp/zxac102 | Purpose of the study not relevant |
| 92 | Jones PH. New insights into carcinogenesis from normal esophagus. Cancer Res. 2023;83(7). http://doi.org/10.1158/1538-7445.AM2023-PL03-01 | Animal experiment |
| 93 | Feng LH, Bu KP, Ren S, Yang Z, Li BX, Deng CE. Nomogram for predicting risk of digestive carcinoma among patients with type 2 diabetes. Diabetes, Metabolic Syndrome and Obesity. 2020;13:1763-70. http://doi.org/10.2147/DMSO.S251063 | Unrelated drug treatment |
| 94 | Sun B, Karin M. Obesity, inflammation, and liver cancer. J Hepatol. 2012;56(3):704-13. http://doi.org/10.1016/j.jhep.2011.09.020 | Review |
| 95 | Ryan AM, Duong M, Healy L, Ryan SA, Parekh N, Reynolds JV, et al. Obesity, metabolic syndrome and esophageal adenocarcinoma: Epidemiology, etiology and new targets. Cancer Epidemiol. 2011;35(4):309-19. http://doi.org/10.1016/j.canep.2011.03.001 | Review |
| 96 | Kashfi K, Rosen CL, Aslan M. Obesity, type-2 diabetes and cancer: Mechanistic insights. Critical Reviews in Oncogenesis. 2019;24(3):285-306. http://doi.org/10.1615/CritRevOncog.2019032959 | Review |
| 97 | Fung BM, David J. Pagetoid Spread of Esophageal Adenocarcinoma. Am J Gastroenterol. 2022;117(10):S1585. http://doi.org/10.14309/01.ajg.0000866092.03697.09 | Case report |
| 98 | Hu WS, Lin CL. Patients with diabetes with and without sodium-glucose cotransporter-2 inhibitors use with incident cancer risk. J Diabetes Complicat. 2023;37(5). http://doi.org/10.1016/j.jdiacomp.2023.108468 | Unrelated drug treatment |
| 99 | Xu S, Xue Y. Pediatric obesity: Causes, symptoms, prevention and treatment (Review). Exp Ther Med. 2016;11(1):15-20. http://doi.org/10.3892/etm.2015.2853 | Review |
| 100 | Nagpal SJS, Macaron C, Alkhouri N. Portal Hypertensive Gastric Polyps Presenting as Iron Deficiency Anemia in a Cirrhotic Patient. Am J Gastroenterol. 2014;1092:S362. http://doi.org/10.14309/00000434-201410002-01225 | Population not relevant |
| 101 | Koshizaka M, Ishibashi R, Maeda Y, Ishikawa T, Maezawa Y, Takemoto M, et al. Predictive model and risk engine web application for surgical site infection risk in perioperative patients with type 2 diabetes. Diabetol Int. 2022;13(4):657-64. http://doi.org/10.1007/s13340-022-00587-w | Purpose of the study not relevant |
| 102 | Loomans-Kropp HA, Chaloux M, Richmond E, Umar A. PROTON PUMP INHIBITOR AND STATIN USE DECREASE RISK OF ESOPHAGEAL ADENOCARCINOMA AMONG INDIVIDUALS WITH BARRETT'S ESOPHAGUS: A SEER-MEDICARE ANALYSIS. Gastroenterology. 2020;1581(6):S87-8. | Unrelated drug treatment |
| 103 | Iyer PG, Borah BJ, Heien H, Chak A. Rates and predictors of progression to adenocarcinoma in a large population based barrett's esophagus cohort. Gastroenterology. 2014;146(5):S121-2. http://doi.org/10.1016/S0016-5085(14)60437-6 | Population not relevant |
| 104 | Krishnamoorthi R, Borah B, Heien H, Das A, Chak A, Iyer PG. Rates and predictors of progression to esophageal carcinoma in a large population-based Barrett's esophagus cohort. Gastrointest Endosc. 2016;84(1):40. http://doi.org/10.1016/j.gie.2015.12.036 | Population not relevant |
| 105 | Ahmed ZSO, Golovoy M, Abdullah Y, Ahmed RSI, Ping Dou Q. Repurposing of metformin for cancer therapy: Updated patent and literature review. Recent Pat Anti-Canc. 2021;16(2):161-86. http://doi.org/10.2174/1574892816666210615163417 | Review |
| 106 | Deb A, Deshmukh B, Ramteke P, Bhati FK, Bhat MK. Resistin: A journey from metabolism to cancer. Transl Oncol. 2021;14(10). http://doi.org/10.1016/j.tranon.2021.101178 | Review |
| 107 | Agrawal K, Markert RJ, Krishnamurthy P, Agrawal S. Risk factors for adenocarcinoma and squamous cell carcinoma of the esophagus and lung. Am J Gastroenterol. 2016;111:S201. http://doi.org/10.1038/ajg.2016.356 | Purpose of the study not relevant |
| 108 | Kambhampati S, Xu Y, Meltzer S. Risk factors for progression of Barrett's esophagus to high-grade dysplasia and esophageal carcinoma. Am J Gastroenterol. 2016;111:S205-6. http://doi.org/10.1038/ajg.2016.356 | Population not relevant |
| 109 | Wang L, Wu Y, Ma J, Li X, Chen Z, Xu W. Risk of malignant tumors in chinese patients with type 2 diabetes mellitus: A retrospective cohort study based on diabetes management data in Jiaxing city, Zhejiang province. Tumor. 2019;39(7):548-57. http://doi.org/10.3781/j.issn.1000-7431.2019.22.083 | Unrelated drug treatment |
| 110 | Deng B, Tang X, Wang Y. Role of microRNA-129 in cancer and non-cancerous diseases (Review). Exp Ther Med. 2021;22(3). http://doi.org/10.3892/etm.2021.10350 | Review |
| 111 | Oparin AA, Oparin AG, Kudriavtsev AA. ROLE OF OXIDATIVE STRESS IN FORMATION MECHANISMS OF GASTROESOPHAGEAL REFLUX DISEASE IN PATIENTS WITH DIABETES MELLITUS TYPE II AND ITS CORRECTION. New Armen Med J. 2018;12(3):67-73. | Population not relevant |
| 112 | Wan X, Liao J, Lai H, Zhang S, Cui J, Chen C. Roles of microRNA-192 in diabetic nephropathy: the clinical applications and mechanisms of action. Front Endocrinol. 2023;14. http://doi.org/10.3389/fendo.2023.1179161 | Review |
| 113 | Diamant M, Van Gaal L, Stranks S, Guerci B, MacConell L, Haber H, et al. Safety and efficacy of once-weekly exenatide compared with insulin glargine titrated to target in patients with type 2 diabetes over 84 weeks. Diabetes Care. 2012;35(4):683-9. http://doi.org/10.2337/dc11-1233 | Unrelated drug treatment |
| 114 | Gupta R, Ratan A, Rajesh C, Chen R, Kim HL, Burhans R, et al. Sequencing and analysis of a South Asian-Indian personal genome. Bmc Genomics. 2012;13(1). http://doi.org/10.1186/1471-2164-13-440 | Purpose of the study not relevant |
| 115 | Dr CCLJ, Montero-Benavides MO, Yarza-Fernández J, Lega-rreta-Holguín J, Flores-Rosillo MA. Severe hypoglycemia in patients with diabetes mellitus type 2 and normal creatinine serum values. Medicina Interna De Mexico. 2017;33(6):739-45. http://doi.org/10.24245/mim.v33i6.1422 | Purpose of the study not relevant |
| 116 | Park JW, Lee JH, Park YH, Park SJ, Cheon JH, Kim WH, et al. Sex-dependent difference in the effect of metformin on colorectal cancer-specific mortality of diabetic colorectal cancer patients. World J Gastroentero. 2017;23(28):5196-205. http://doi.org/10.3748/wjg.v23.i28.5196 | Population not relevant |
| 117 | Abdelghani L, Modha K, Albaddawi E, Subramanian S. Sodium-wasting nephropathy caused by cisplatin in esophageal cancer. Journal of Supportive Oncology. 2008;6(7):305-6. | Population not relevant |
| 118 | Lin SH. Statin Use and Chemoradiation in Esophageal Squamous Cell Carcinomas: Ready for Prime Time? J Thorac Oncol. 2023;18(8):970-1. http://doi.org/10.1016/j.jtho.2023.05.020 | Unrelated drug treatment |
| 119 | Harder EM. Stereotactic Body Radiation Therapy for Early Stage Lung Cancer: a Report on Efficacy and Toxicity.; 2015. | Case report |
| 120 | Monami M, Lamanna C, Balzi D, Marchionni N, Mannucci E. Sulphonylureas and cancer: A case-control study. Acta Diabetol. 2009;46(4):279-84. http://doi.org/10.1007/s00592-008-0083-2 | Unrelated drug treatment |
| 121 | Tomillero A, Moral MA. Summary. Methods and Findings in Experimental and Clinical Pharmacology. 2010;32(5):331-88. http://doi.org/10.1358/mf.2010.32.5.1520420 | Purpose of the study not relevant |
| 122 | Korsse SE, Peppelenbosch MP, Van Veelen W. Targeting LKB1 signaling in cancer. Biochimica Et Biophysica Acta - Reviews On Cancer. 2013;1835(2):194-210. http://doi.org/10.1016/j.bbcan.2012.12.006 | Review |
| 123 | Sainero-Alcolado L, Liaño-Pons J, Ruiz-Pérez MV, Arsenian-Henriksson M. Targeting mitochondrial metabolism for precision medicine in cancer. Cell Death Differ. 2022;29(7):1304-17. http://doi.org/10.1038/s41418-022-01022-y | Review |
| 124 | Wiebe N, Ye F, Crumley ET, Bello A, Stenvinkel P, Tonelli M. Temporal Associations among Body Mass Index, Fasting Insulin, and Systemic Inflammation: A Systematic Review and Meta-analysis. Jama Netw Open. 2021;4(3). http://doi.org/10.1001/jamanetworkopen.2021.1263 | Meta-analysis |
| 125 | Bohle W. The 42nd Congress of the German Southwest Society of Internal Medecine: The internist is more than ever needed. Deut Med Wochenschr. 2005;130(49):2809-10. | Conference |
| 126 | Sekino N, Hoshino I, Akutsu Y, Akanuma N, Isozaki Y, Murakami K, et al. The antitumor effect of metformin on esophageal squamous cell carcinoma cells. Dis Esophagus. 2014;27:119A. http://doi.org/10.1111/dote.12287 | Cell experiment |
| 127 | Alvarado CE, Kapcio KC, Lada MJ, Linden PA, Towe CW, Worrell SG. The Effect of Diabetes on Pathologic Complete Response Among Patients With Esophageal Cancer. Semin Thorac Cardiov. 2023;35(2):429-36. http://doi.org/10.1053/j.semtcvs.2021.10.016 | Unable to get the outcome indicate |
| 128 | Wu HD, Zhang JJ, Zhou BJ. The effect of metformin on esophageal cancer risk in patients with type 2 diabetes mellitus: a systematic review and meta‑analysis. Clinical and Translational Oncology. 2021;23(2):275-82. http://doi.org/10.1007/s12094-020-02415-6 | Meta‑analysis |
| 129 | Liu J, Zhang M, Deng D, Zhu X. The function, mechanisms, and clinical applications of metformin: potential drug, unlimited potentials. Arch Pharm Res. 2023;46(5):389-407. http://doi.org/10.1007/s12272-023-01445-2 | Review |
| 130 | Harvey AE, Lashinger LM, Hursting SD. The growing challenge of obesity and cancer: An inflammatory issue.; 2011. p. 45-52. | Review |
| 131 | Tseng C. The Relationship between Diabetes Mellitus and Gastric Cancer and the Potential Benefits of Metformin: An Extensive Review of the Literature. Biomolecules. 2021;11(10227). http://doi.org/10.3390/biom11071022 | Review |
| 132 | Najafi F, Rajati F, Sarokhani D, Bavandpour M, Moradinazar M. The relationship between metformin consumption and cancer risk: An updated umbrella review of systematic reviews and meta-analyses. Int J Preventive Med. 2023;14(1). http://doi.org/10.4103/ijpvm.ijpvm_62_21 | Meta-analysis |
| 133 | Colmers IN, Bowker SL, Johnson JA. Thiazolidinedione use and cancer incidence in type 2 diabetes: A systematic review and meta-analysis. Diabetes and Metabolism. 2012;38(6):475-84. http://doi.org/10.1016/j.diabet.2012.06.003 | Meta-analysis |
| 134 | Tunio MA, AlAsiri M, Fareed MM, Ali NMO. Tongue metastasis as an initial manifestation of distant metastasis in oesophageal adenocarcinoma. Pak J Med Sci. 2014;30(4). http://doi.org/10.12669/pjms.304.4910 | Case report |
| 135 | Sorrell AD, Espenschied CR, Culver JO, Weitzel JN. Tumor protein p53 (TP53) testing and li-fraumeni syndrome: Current status of clinical applications and future directions. Molecular Diagnosis and Therapy. 2013;17(1):31-47. http://doi.org/10.1007/s40291-013-0020-0 | Review |
| 136 | Raskin P, Lewin A, Reinhardt R, Lyness W, Adler J, Ahmed A, et al. Twice-daily dosing of a repaglinide/metformin fixed-dose combination tablet provides glycaemic control comparable to rosiglitazone/metformin tablet. Diabetes, Obesity and Metabolism. 2009;11(9):865-73. http://doi.org/10.1111/j.1463-1326.2009.01062.x | Purpose of the study not relevant |
| 137 | Takata K, Ishii F, Uchida Y, Fukuda H, Yamauchi R, Umeda K, et al. Two cases of hepatocellular carcinoma arising over 20 years after a sustained virologic response following interferon therapy for chronic Hepatitis C. Internal Med. 2020;59(15):1855-60. http://doi.org/10.2169/internalmedicine.4479-20 | Population not relevant |
| 138 | Rahman I, Athar MT, Islam M. Type 2 Diabetes, Obesity, and Cancer Share Some Common and Critical Pathways. Front Oncol. 2020;10. http://doi.org/10.3389/fonc.2020.600824 | Review |
| 139 | Anitha N, Sherene Christina Roshini AM, Aravindha Babu N, Rajesh E. Type of article: Review article title of the article: Microbiome in health and diseased. European Journal of Molecular and Clinical Medicine. 2020;7(4):1486-91. | Review |
| 140 | Papanagnou P, Stivarou T, Tsironi M. Unexploited antineoplastic effects of commercially available anti-diabetic drugs. Pharmaceuticals-Base. 2016;9(2). http://doi.org/10.3390/ph9020024 | Review |
| 141 | Karasic DS, Pearson VE. Urticaria and respiratory distress due to porfimer sodium [4]. Ann Pharmacother. 2000;34(10):1208-9. http://doi.org/10.1345/aph.19352 | Purpose of the study not relevant |
| 142 | Søndergaard CS, Esquivel PN, Dalamaga M, Magkos F. Use of Antihyperglycemic Drugs and Risk of Cancer in Patients with Diabetes. Curr Oncol Rep. 2023;25(1):29-40. http://doi.org/10.1007/s11912-022-01344-7 | Review |
| 143 | Kautzky-Willer A, Thurner S, Klimek P. Use of statins offsets insulin-related cancer risk. J Intern Med. 2017;281(2):206-16. http://doi.org/10.1111/joim.12567 | Unrelated drug treatment |
| 144 | Macnab MR, Staff R, Mckiddie F, Denison AR, Olson S, Ramage E, et al. Variation in bowel uptake during 18F-FDG PET imaging between patients with different cancer indications. Mol Imaging Biol. 2016;18(2):S1456-7. http://doi.org/10.1007/s11307-016-1030-1 | Purpose of the study not relevant |
| 145 | Elliott JA, Reynolds JV. Visceral Obesity, Metabolic Syndrome, and Esophageal Adenocarcinoma. Front Oncol. 2021;11. http://doi.org/10.3389/fonc.2021.627270 | Review |
| 146 | O'Flanagan CH, Smith LA, McDonell SB, Hursting SD. When less may be more: Calorie restriction and response to cancer therapy. Bmc Med. 2017;15(1). http://doi.org/10.1186/s12916-017-0873-x | Unrelated drug treatment |
| 147 | Loomans-Kropp HA, Chaloux M, Richmond E, Umar A. 461 PROTON PUMP INHIBITOR AND STATIN USE DECREASE RISK OF ESOPHAGEAL ADENOCARCINOMA AMONG INDIVIDUALS WITH BARRETT'S ESOPHAGUS: A SEER-MEDICARE ANALYSIS. Gastroenterology. 2020;158(6):87-8. http://doi.org/10.1016/S0016-5085(20)30897-0 | Unrelated drug treatment |
| 148 | Charette N, Leclercq IA. Why should the gastroenterologist bother about obesity? An oncologic point of view. Acta Gastro-Ent Belg. 2010;73(4):504-9. | Review |
