# Supplementary material for: Association of metformin use with risk and survival outcome of esophageal cancer in patients with diabetes: A systematic review and meta-analysis
Source: PLoS One. 2025 Jan 7;20(1):e0310687. doi: 10.1371/journal.pone.0310687 (PMC11706492; doi:10.1371/journal.pone.0310687)
Supplement: S4 Table — (DOCX) [file pone.0310687.s005.docx]

Included articles

| No. | Reference |
| --- | --- |
| 1 | Wang Q, Santoni G, Ness-Jensen E, Lagergren J, Xie S. Association Between Metformin Use and Risk of Esophageal Squamous Cell Carcinoma in a Population-Based Cohort Study. Am J Gastroenterol. 2020;115(1):73-78. DOI: 10.14309/ajg.0000000000000478 |
| 2 | Tseng C. Metformin and esophageal cancer risk in Taiwanese patients with type 2 diabetes mellitus. ONCOTARGET. 2017;8(12):18802-18810. DOI: 10.18632/oncotarget.13390 |
| 3 | Sung JJ, Ho JM, Lam AS, Yau ST, Tsoi KK. Use of metformin and aspirin is associated with delayed cancer incidence. Cancer Epidemiol. 2020;69:101808. Epub 2020-12-1. doi: 10.1016/j.canep.2020.101808. |
| 4 | Tsilidis KK, Capothanassi D, Allen NE, Rizos EC, Lopez DS, Van Veldhoven K, et al. Metformin does not affect cancer risk: A cohort study in the U.K. clinical practice research datalink analyzed like an intention-to-treat trial. Diabetes Care. 2014;37(9):2522-2532. Epub 2014-1-1. doi: 10.2337/dc14-0584. |
| 5 | Lee M, Hsu C, Wahlqvist ML, Tsai H, Chang Y, Huang Y. Type 2 diabetes increases and metformin reduces total, colorectal, liver and pancreatic cancer incidences in Taiwanese: a representative population prospective cohort study of 800,000 individuals. BMC Cancer. 2011;11(20). doi: 10.1186/1471-2407-11-20. |
| 6 | Murff HJ, Roumie CL, Greevy RA, Hackstadt AJ, McGowan LED, Hung AM, et al. Metformin use and incidence cancer risk: evidence for a selective protective effect against liver cancer. Cancer Causes Control. 2018;29(9):823-832. doi: 10.1007/s10552-018-1058-4. |
| 7 | De Jong RG, Burden AM, De Kort S, Van Herk-Sukel MP, Vissers PA, Janssen PK, et al. No decreased risk of gastrointestinal cancers in users of metformin in the Netherlands; A time-varying analysis of metformin exposure. Cancer Prev Res (Phila). 2017;10(5):290-297. Epub 2017-1-1. doi: 10.1158/1940-6207.CAPR-16-0277 |
| 8 | Ruiter R, Visser LE, Van Herk-Sukel MPP, Coebergh JWW, Haak HR, Geelhoed-Duijvestijn PH, et al. Lower risk of cancer in patients on metformin in comparison with those on sulfonylurea derivatives: Results from a large population-based follow-up study. Diabetes Care. 2012;35(1):119-124. Epub 2012-1-1. doi: 10.2337/dc11-0857 |
| 9 | Oh TK, Song IA. Metformin Use and the Risk of Cancer in Patients with Diabetes: A Nationwide Sample Cohort Study. Cancer Prev Res (Phila). 2020;13(2):195-202. Epub 2020-2-1. doi: 10.1158/1940-6207.CAPR-19-0427. |
| 10 | Becker C, Meier CR, Jick SS, Bodmer M. Case-control analysis on metformin and cancer of the esophagus. Cancer Causes Control. 2013;24(10):1763-70. Epub 2013-10-1. doi: 10.1007/s10552-013-0253-6. |
| 11 | Valent F. Diabetes mellitus and cancer of the digestive organs: An Italian population-based cohort study. J Diabetes Complications. 2015;29(8):1056-1061. Epub 2015-1-1. doi: 10.1016/j.jdiacomp.2015.07.017. |
| 12 | Cheng KC, Chen YL, Lai SW, Tsai PY, Sung FC. Risk of esophagus cancer in diabetes mellitus: a population-based case-control study in Taiwan. BMC Gastroenterol. 2012;12:177. Epub 2012-12-12. doi: 10.1186/1471-230X-12-177. |
| 13 | Van De Voorde L, Janssen L, Larue R, Houben R, Buijsen J, Sosef M, et al. Can metformin improve 'the tomorrow' of patients treated for oesophageal cancer? Eur J Surg Oncol. 2015;41(10):1333-9. Epub 2015-10-1. doi: 10.1016/j.ejso.2015.05.012. |
| 14 | He HH, Fu JH, Hao ZX, Wu HF, Zhong Q, Wang F, et al. Impact of metformin on survival outcome of esophageal squamous cell carcinomas patients undergoing surgical resection: A multicenter retrospective study. J Thorac Dis. 2020;12(3):830-838. Epub 2020-1-1. doi: 10.21037/jtd.2019.12.98. |
| 15 | Spierings L, Van Laarhoven H, Lagarde S, Van Oijen M, Gisbertz S, Wilmink J, et al. Metformin use during treatment of resectable esophageal cancer patients is not associated with better outcomes. United European Gastroenterol J. 2015;3(5):A483. Epub 2015-1-1. doi: 10.1177/2050640615601623. |
| 16 | Wang QL, Santoni G, Lagergren J. Diabetes, metformin use, and survival in esophageal cancer: a population-based cohort study. JNCI Cancer Spectr. 2023;7(4)Epub 2023-7-3. doi: 10.1093/jncics/pkad043. |
