# Supplementary material for: Association of metformin use with risk and survival outcome of esophageal cancer in patients with diabetes: A systematic review and meta-analysis
Source: PLoS One. 2025 Jan 7;20(1):e0310687. doi: 10.1371/journal.pone.0310687 (PMC11706492; doi:10.1371/journal.pone.0310687)
Supplement: Attachment — Submitted filename: review.doc [file pone.0310687.s006.doc]

First, I would like to praise your effort in this metanalysis and data collection and there is one main point for me need clarification

**Heterogenicity test index in association of metformin with the EC incidence in patients with diabetes is near the high index and the subgroup analysis did not eliminate its effect and did not answer questions like how long did patients suffer from diabetes before EC and how long did they use metformin before diagnosis by EC**
