# Supplementary material for: Association of metformin use with risk and survival outcome of esophageal cancer in patients with diabetes: A systematic review and meta-analysis
Source: PLoS One. 2025 Jan 7;20(1):e0310687. doi: 10.1371/journal.pone.0310687 (PMC11706492; doi:10.1371/journal.pone.0310687)
Supplement: Attachment — Submitted filename: Response to Reviewers.doc [file pone.0310687.s007.doc]

Reviewer #2

First, I would like to praise your effort in this metanalysis and data collection and there is one main point for me need clarification

Heterogenicity test index in association of metformin with the EC incidence in patients with diabetes is near the high index and the subgroup analysis did not eliminate its effect and did not answer questions like how long did patients suffer from diabetes before EC and how long did they use metformin before diagnosis by EC

**Author response:** Thanks for the suggestion.

Despite significant heterogeneity in this study, we performed a sensitivity analysis (excluding studies one by one) and determined that removing any one study did not affect the results. Our subgroup analysis indicates that the selection of reference materials, time bias, and confounding factors such as BMI and smoking may be sources of heterogeneity. We assessed the quality of evidence using GRADE, suggesting that our results possess a certain level of credibility. As a result of the limitations of the data in the included literatures, we were unable to determine the duration of diabetes before the diagnosis of esophageal cancer. We emailed the authors asking for details but received no response. We included the duration of metformin use in Table 1.

**Reviewer #1:** 1.Please indicate reference number for each study in table 1. Please provide the breakdown, indicating the number of patients/ participants on Metformin vs no Metformin. Currently on table 1, there is only one group as NM (Non-Metformin).

**Author response:** Thanks for the suggestion.

We indicated the reference number for each study in table 1. We provided the breakdown, indicating the number of patients taking metformin and those not taking metformin.

2.What was the absolute number of new cases of esophageal cancer in the group exposed to Metformin and non-Metformin, in each study where incidence was the outcome of interest and combined.

**Author response:** We updated the absolute number of new esophageal cancer cases and incidence rate in the group exposed to Metformin and non-Metformin (Table 1).

3.What was the stage of the disease and histopathology in each study where a therapeutic intervention was done.

**Author response:** We supplemented the disease stage and histopathological information (Table 1). The patients with esophageal cancer were primarily in the middle to advanced stages in the included studies. Our results indicate that metformin prolongs overall survival in patients with diabetes and esophageal cancer (especially esophageal squamous cell carcinoma). Details can be seen in Table 1.

4.Authors have provided significant statistical differences without any absolute numbers of an outcome/ event related to esophageal cancer or clinical characteristics of the patients exposed to Metformin or no Metformin, sulphonylureas or no sulphonylureas.

**Author response:** We added absolute numbers of outcomes/events or clinical features related to esophageal cancer (Table 1: NO. of new cases of EC; Incidence rate; No. of deaths among patients with EC; Mortality rate).

5.Given the huge sample size in the analyzed studies and without detailed clinical information of the participants, incidence/ extent of esophageal cancer it is challenging to make a sense of statistical values provided in the manuscript.

**Author response:** We provided additional detailed clinical information for participants, including the number of new esophageal cancer cases, incidence rate, number of esophageal cancer deaths, mortality rate, disease staging, and tissue type (Table 1). We reached out to the authors of the included studies to obtain more patient information but received no response.
